# Chorea

**Published:** 1875-10

**Authors:** Henry Nichell


ART. II.—*Chorea or St. Vitus' Dance.* By Henry Nichell, M. D.

From December 30th, 1874, to September 8th, of the present year, I have had under my care four cases of St. Vitus' Dance, in which the subjoined combination has been prescribed, leading to a speedy recovery :

℞

Strychnia Sulph. ............................gr. ss.
Acetic Acid, dilut............................q. s.
Pyrophosphate of Iron.........................ʒiss
Cinnamon Water ...............................℥iij
Extr. Belladonna..............................gr. ij
Syr. Orange peel .............................℥j
Fowler's Solution ............................ʒiss.

M. Sig—A teaspoonful three times daily after meals. (To be well shaken).

Case I.—L. G., a girl eight years old, had the disease steady for five weeks. The above remedy was taken for about three weeks, when she appeared entirely free from the malady.

Case II.—C. S., a girl of the same age, had suffered from chorea for about two months; she had been attended by an experienced physician during that time, but as no benefit was derived from treatment, the attendant abstained from further advice in the case. After some time I was called, and ordered the above prescription. In the course of three weeks the child recovered.

Case III.—C. W., a girl aged six years, had chorea for two weeks, when the same medicine was administered, resulting in recovery at the end of two weeks.

Case IV.—G. E., a boy six years old, had suffered from a severe attack of articular rheumatism of most joints from the latter part of July up to August 9th, when I was summoned. The patient then had Scarlatina Anginosa: severe vomiting, profuse diarrhœa, and swelling of some of the joints were present. Soon afterward symptoms of Cerebro-Spinal Meningitis set in, and on the twentieth of August twitchings of the muscles of the face came on. Shortly afterward almost all the voluntary muscles were affected. Articulation was entirely abolished and deglutition was seriously interfered with. My friend, Dr. E. Tobie, met me in consultation on the twenty-first of August, and pronounced the case a very unfavorable one, since the meningeal affection had not subsided, the abdomen being tympanitic and watery diarrhœa having again returned. After some days, however, the latter phenomena subsided. On the twenty-fourth of August I prescribed the mixure administered in the other cases of chorea, and in less than eight days the little patient had fully recovered.

As regards the composition of the remedy which I have used in these four cases, some one of its ingredients may probably be dispensed with—perhaps belladonna. But what I desire, is to call the attention of the profession to this combination (which, of course, must be modified and adapted to age, constitution, etc., of the patient), and to ask that it receive a fair trial in so troublesome a disease as Chorea.

